# CD4 ^+^ CD11b ^+^ T cells infiltrate and aggravate the traumatic brain injury depending on brain‐to‐cervical lymph node signaling

**DOI:** 10.1111/cns.14673

**Published:** 2024-03-11

**Authors:** Weiwei Jiang, Xuanhui Liu, Yupeng Chen, Mingqi Liu, Jiangyuan Yuan, Meng Nie, Yibing Fan, Di Wu, Yu Qian, Zhuang Sha, Shiying Dong, Chenrui Wu, Tao Liu, Jinhao Huang, Jianning Zhang, Chuang Gao, Rongcai Jiang

**Affiliations:** ^1^ Department of Neurosurgery General Hospital of Tianjin Medical University Tianjin China; ^2^ State Key Laboratory of Experimental Hematology Tianjin China; ^3^ Tianjin Neurological Institute, Key Laboratory of Post‐Neuroinjury Neurorepair and Regeneration in Central Nervous System Tianjin Medical University General Hospital, Ministry of Education Tianjin China; ^4^ Department of Neurosurgery Tianjin First Central Hospital Tianjin China

**Keywords:** brain‐to‐cervical lymph node signaling, CD4^+^ T cell, immunoregulation, traumatic brain injury

## Abstract

**Aim:**

We aim to identify the specific CD4^+^ T‐cell subtype influenced by brain‐to‐CLN signaling and explore their role during the acute phase of traumatic brain injury (TBI).

**Method:**

Cervical lymphadenectomy or cervical afferent lymphatic ligation was performed before TBI. Cytokine array and western blot were used to detect cytokines, while the motor function was assessed using mNss and rotarod test. CD4^+^ T‐cell subtypes in blood, brain, and CLNs were analyzed with Cytometry by time‐of‐flight analysis (CyTOF) or fluorescence‐activated cell sorting (FACS). Brain edema and volume changes were measured by 9.4T MRI. Neuronal apoptosis was evaluated by terminal deoxynucleotidyl transferase‐mediated dUTP nick end labeling (TUNEL) staining.

**Results:**

Cervical lymphadenectomy and ligation of cervical lymphatic vessels resulted in a decreased infiltration of CD4^+^ T cells, specifically CD11b‐positive CD4^+^ T cells, within the affected region. The population of CD4^+^CD11b^+^ T cells increased in ligated CLNs, accompanied by a decrease in the average fluorescence intensity of sphingosine‐1‐phosphate receptor‐1 (S1PR1) on these cells. Administration of CD4^+^CD11b^+^ T cells sorted from CLNs into the lateral ventricle reversed the attenuated neurologic deficits, brain edema, and lesion volume following cervical lymphadenectomy.

**Conclusion:**

The infiltration of CD4^+^CD11b^+^ T cells exacerbates secondary brain damage in TBI, and this process is modulated by brain‐to‐CLN signaling.

## Graphical abstract

Traumatic brain injury (TBI) elicits damage signals that are conveyed via the meningeal lymphatic vessels(mLVs) to cervical lymph nodes (CLNs), prompting the egress of CD4^+^CD11b^+^ T cells from the CLNs. Subsequently, these cells traverse the compromised blood–brain barrier to infiltrate the impaired brain aggravate the damage.

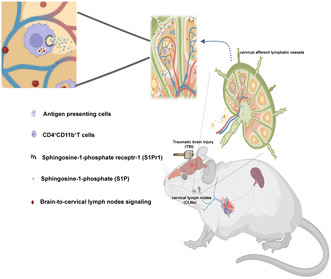

## 1 INTRODUCTION

Traumatic brain injury (TBI) involves primary damage such as neuron necrosis, diffuse axonal injury, and bleeding caused by external forces. These primary injuries result in secondary damage, including immune cell infiltration, neuroinflammation, brain edema, oxidative stress, and neuronal apoptosis,^1^ which contribute to high mortality and disability rates.^2^ Extensive research has been dedicated to understanding the intricate mechanisms of TBI, particularly the role of peripheral immune cells in secondary brain damage.^1^, ^3^, ^4^

Existing studies have linked CD4^+^ T‐cell infiltration and neuroinflammation, highlighting the diverse functions of CD4^+^ T‐cell subsets in immune regulation, including Th0, Th1, Th2, Th17, and Treg cells.^5^ Different subsets of CD4^+^ T‐cells can either contribute to autoimmune disorders or help mitigate post‐traumatic degeneration.^6^, ^7^, ^8^ Despite the awareness of the potential impact of systemic CD4^+^ T‐cell responses on brain injury and recovery post‐TBI,^9^, ^10^ the precise mechanisms by which the injured brain initiates signals to facilitate CD4^+^ T‐cell infiltration remain poorly understood. Further investigation is needed to understand the specific characteristics and mechanisms of CD4^+^ T‐cell response in the injured brain.

The extracellular solutes in brain interstitial fluid (ISF) are drained to cervical lymph nodes (CLNs) through meningeal lymphatic vessels (mLVs).^11^, ^12^ The identification of the brain‐mLVs‐ CLN drainage pathway has considerably enhanced our comprehension of brain‐to‐CLN signaling, offering innovative insights into the infiltration of CD4^+^ T cells in brain subsequent to TBI. This signaling concept was initially defined by Lo and Hayakawa in 2019 and pertains to the activation of CLN by the injury products of the central nervous system (CNS), including macromolecules and cellular fragments.^13^ Modulating these signals offer a potential therapeutic approach for CNS disorders.^14^, ^15^ Actually, research on experimental allergic encephalomyelitis (EAE) has shown that surgical excision of CLNs reduces the infiltration of T cells into the brain and relapse severity.^16^ This observation indicates that the signaling emanating from the brain to the CLN may be responsible for triggering systemic T‐cell responses. Cannulation studies have consistently demonstrated a higher rate of egress for CD4^+^ T cells than CD8^+^ T cells.^17^, ^18^ The empirical evidence from these studies indicates a plausible link between brain‐to‐CLN signaling and the infiltration of CD4^+^ T cells into the brain after TBI.

Our research aims to determine the specific subtype of CD4^+^ T cells affected by brain‐to‐CLN signaling during the acute phase of TBI and their functional contributions. To investigate this, surgical interventions, including cervical lymphadenectomy and cervical afferent lymphatic ligation, were performed to assess their impact on CD4^+^ T‐cell infiltration in the brain.

## 2 MATERIALS AND METHODS

### 2.1 Reagents and antibodies

See the Table S1 for details.

### 2.2 Mice

Male C57BL/6 mice (6–8 weeks old, weighing 24–27 g) obtained from Vital River Laboratory Animal Technology Co, Ltd, Beijing, China, were housed in clean environments with 12‐h light–dark cycles in accordance with the approved protocol of the Tianjin Medical University Animal Ethics Committee (Tianjin, China). Animals were randomly assigned to different treatment groups.

### 2.3 Controlled cortical impact (CCI) mice model

The CCI model was employed in accordance with previously established protocols.^19^ In summary, a circular impact tip measuring 2 mm in diameter was directed vertically toward the surface of the dura mater. This was achieved with parameters set at 5.5 m/s velocity, 2.0 mm depth, and 100 ms duration. The sham group underwent an identical process except for the impact procedure.

### 2.4 Cervical afferent lymphatic ligation and cervical lymphadenectomy

Mice were anesthetized, their hair cleared, and their skin sterilized. A small incision was made at the neck midline to expose the CLNs. CLNs were removed, or the cervical lymphatic vessels were ligated while protecting nearby arteries and nerves. Sham mice underwent the same procedure without any intervention. The incision was closed, and postoperative care was provided.

### 2.5 Intracerebroventricular injection (icv)

Mice were anesthetized and positioned in a stereotaxic apparatus. We administered injections (10 μL each) of target solution (saline solution, 2% Evans Blue solution, brain ISF, or cell suspension with a concentration of 2.5 × 10 ^7^ cells/mL) into the lateral ventricle (0.5 mm posterior to the bregma, ±1.0 mm lateral to the midline, and 2.0 mm ventral to the brain surface). Ketoprofen (2 mg/kg) was given subcutaneously post‐surgery, and mice were closely monitored.

### 2.6 Rotarod test

Mice in each group underwent exercise endurance assessment using the Rotarod apparatus, with 5 min acclimation period at 5 rpm and a gradual acceleration to 40 rpm in 90 s. The mice were then tested for 5 min, and the time they fell or completed five turns was recorded. If a mice completed the full 5 min without falling, a falling time of 300 s was recorded. Three rounds of testing were performed with 30 min interval between each round, and the average falling time was calculated.

### 2.7 Modified neurological severity score (mNSS)

Neurologic impairment in TBI mice was assessed using the mNSS,^20^ which evaluated motor, sensory, and reflex functions. The scoring scale ranged from 0 to 18 (0 indicating a standard score and 18 indicating the maximum score). Sensorimotor function was assessed at 1, 3, and 7 days after CCI, with higher mNSS scores indicating poorer function.

### 2.8 Extraction of evans blue (EB)

Serum was collected through centrifugation and then added to wells in a 96‐well plate for EB concentration measurement. A standard curve was prepared using diluted EB solution in serum. The optical density at 620 nm was measured using an ELISA reader to calculate the actual EB concentration in each sample based on the standard curve.

### 2.9 Fluorescence‐activated cell sorting (FACS)

The procedure followed previously published research.^21^ Briefly, the tissue was washed, cut, and then dissociated by mixing with collagenase IV. After centrifugation and resuspension in Percoll‐PBS, myelin sheaths and fluid were removed. The cells were washed, suspended in 1% BSA‐PBS, distributed into test tubes, and stained with fluorescent‐labeled antibodies. Flow cytometry equipment and FlowJo software were used for analysis.

### 2.10 Cytometry by time‐of‐flight analysis (CyTOF)

Brain tissue was dissociated into individual cells using the Mice Brain Tissue Cell Dissociation Kit.^22^ Fixation and labeling with cisplatin were performed. Cell counting and barcoding were achieved. The cells were then incubated with labeled extracellular antibodies. Fixation, washing, and resuspension were followed. Methanol, iridium, and EQ_beads were added. The cells were filtered and analyzed using the Helios system. Data analysis and visualization were done with R software to identify cell populations based on marker expression. Data from three independent mice were pooled together for analysis.

### 2.11 Extraction of interstitial fluid (ISF)

Brain (cerebellum removed) was rapidly harvested after killing by complete perfusion of cold PBS buffers into the heart at 6 h post‐TBI. Gently grind the finely chopped brain using a rubber grinding stick and a 70 μm cell filter. As the tissue was grinding, 1.5 mL cold diluent (PBS + 1:25 protease inhibitor) was gradually added. After centrifugation at 250 g at 4°C for 5 min, the supernatant was extracted and then centrifuged at 10,000 g 4°C for 15 min. The supernatant was extracted again as diluted ISF and filtered by a 0.22 μm filter. All ISF extraction operations were performed on ice.

### 2.12 Western blot

The methodology for conducting western blot analysis was guided by previously published literature.^23^, ^24^ In brief, the procedure involves the collection of protein samples derived from damaged brain tissue, followed by protein electrophoresis and PVDF membrane transfer. Subsequently, the membrane is blocked using a 5% skim milk solution for of 2 h. The appropriate concentration of the primary antibody to be utilized is determined by referring to the antibody datasheet.

### 2.13 Magnetic resonance imaging (MRI) scan

A Bruker BioSpec 94/30 USR MRI scanner with 9.4T field intensity was used to scan the head. Edematous brain tissue and liquefied foci appeared as long signals in T2‐weighted scans compared to normal brain tissue. Analysis was done using 3D Slicer software with a defect lesion threshold set at 0–10,000 and an edema lesion threshold above 13,000.

### 2.14 Immunohistochemistry, imaging, and quantification^23^

After blocking, the tissue was incubated overnight at 4°C with a diluted primary antibody. The sections were washed and incubated with a fluorescent‐labeled secondary antibody. After washing, the sections were sealed with a DAPI‐containing sealant. For TUNEL staining, a mixture of TDT enzyme and fluorescent‐labeling solution was used according to the instructions, followed by incubation and sealing. Finally, the sections were observed and photographed using a fluorescent inverted microscope, and ImageJ software was used for analysis.

### 2.15 Data analysis

Data were expressed as mean ± SD. The normality of the data distribution was assessed using the Shapiro–Wilk test. An unpaired two‐tailed Student's t‐test was employed. For comparisons among multiple groups, one‐way or two‐way variance analysis (ANOVA) was conducted, followed by Tukey's post hoc test. Statistical significance was considered at *p* < 0.05. Data analysis and plotting were performed using GraphPad Prism 9.0.

## 3 RESULTS

### 3.1 Cervical lymphadenectomy improved motor function and reduced the expression of inflammatory cytokines

**FIGURE 1:**
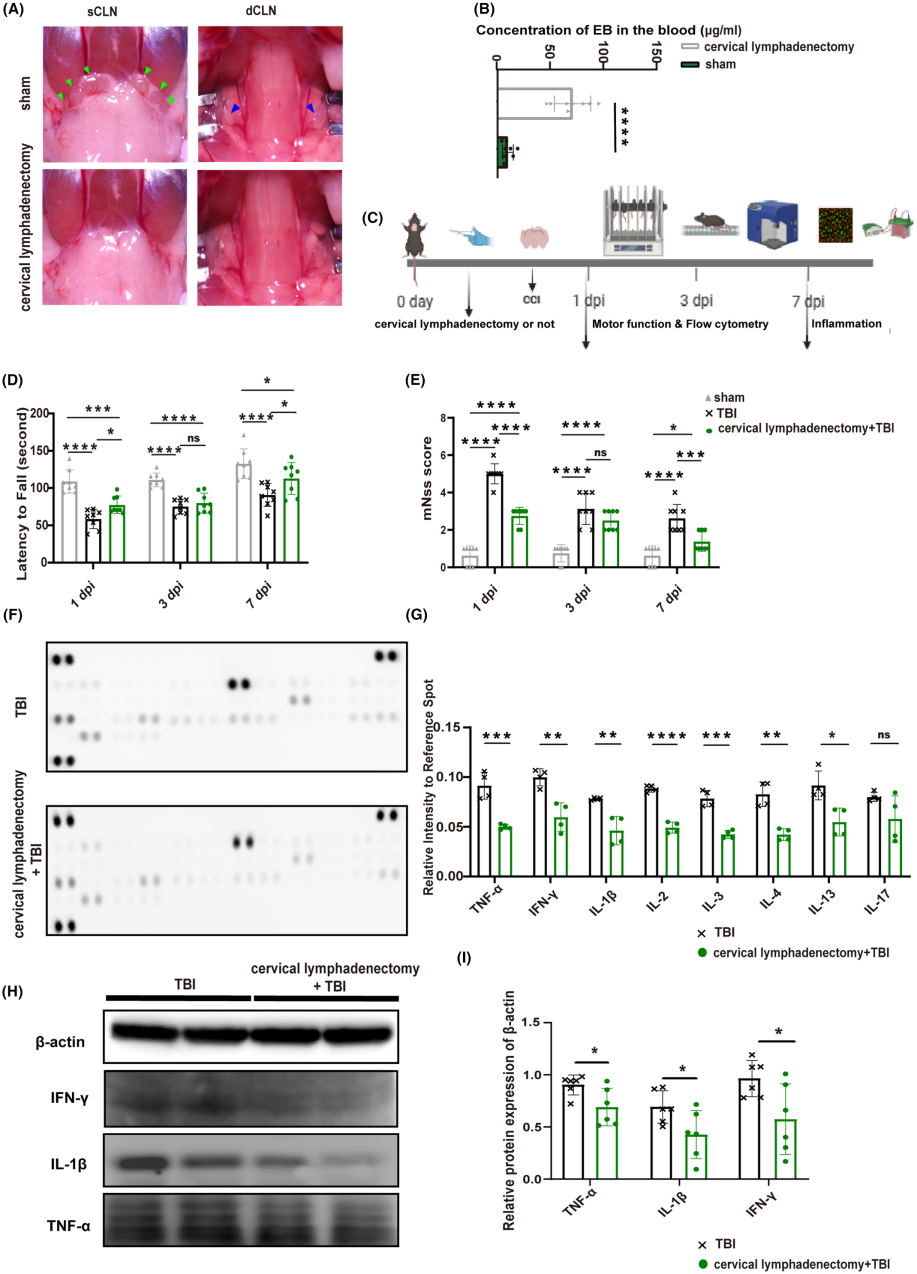
The motor function and expression of cytokines after cervical lymphadenectomy. (A) Display of cervical lymphadenectomy; green arrows indicate sCLNs; blue arrows indicate dCLNs. (B) The concentration of Evans Blue in peripheral blood in mice with or without CLNs (*n* = 8). (C) Basic characteristics of this part. (D) Statistical analysis of the Rotarod experiment (*n* = 8). (E) The results of mNSS in each group (*n* = 8). (F) The exposure image displays inflammatory cytokines using the mice cytokine array at 7 dpi. (G) Statistical analysis of the pixel density of elevated cytokines at 7 dpi. The samples were derived from a mixture of three independent mice, and each inflammatory factor detection point represents one experiment (*n* = 4). (H) Representative western blots of β‐actin, IFN‐γ, TNF‐α, and IL‐1β. (I) Quantification of relative protein expression normalized to the optical density of β‐actin (*n* = 6). All data are shown as mean ± SD. *p < 0.05, **p < 0.01, ***p < 0.001, and ****p < 0.0001. dCLNs, deep cervical lymph nodes; dpi, day post‐injury; IFN‐γ, interferon‐gamma; IL, interleukin; mNSS, modified neurological severity score; sCLNs, superficial cervical lymph nodes; TNF‐α, tumor necrosis factor‐alpha.

To study the role of CLNs in TBI, we surgically removed both deep and superficial CLNs in mice (Figure 1A) and injected a 2% EB solution into the lateral ventricle. The decrease of EB concentration in peripheral blood 2 h later after cervical lymphadenectomy indicated that CLNs serve as relay stations connecting the brain and the periphery (Figure 1B). The specific experimental procedure for this section is illustrated in Figure 1C. Cervical lymphadenectomy before TBI enhanced motor function and neural function scores in mice compared to those without lymphadenectomy at 1 and 7 days post‐injury (dpi) (Figure 1D,E). Based on existing literature indicating the peak infiltration of CD4^+^ T cells at 7 dpi period,^25^, ^26^ we conducted the detection of inflammatory factors in brain tissue at 7 dpi. The results showed that cervical lymphadenectomy led to decreased levels of inflammatory cytokines, including tumor necrosis factor‐alpha (TNF‐α), interferon‐gamma (IFN‐γ), and interleukin‐1β (IL‐1β), T‐cell‐related cytokines such as IL‐4 also showed a similar trend (Figure 1F,G). Consistent findings were observed in the western blot analysis at 7 dpi (Figure 1H,I).

### 3.2 Cervical lymphadenectomy reduces the infiltration of CD4 ^+^ T cells expressing high levels of CD11b into the brain post‐TBI

**FIGURE 2:**
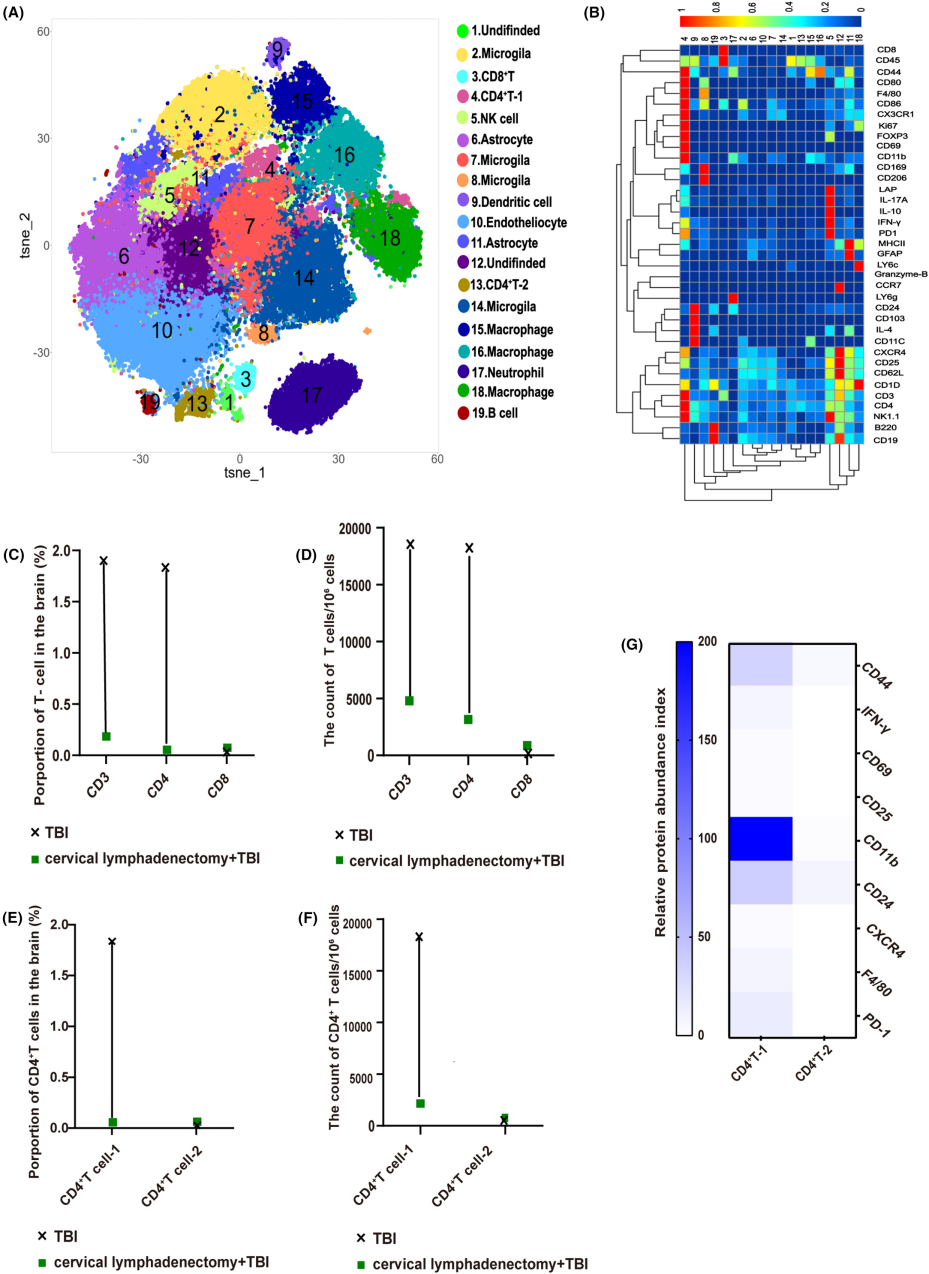
Cytometry by time‐of‐flight analysis of the brain at 7 dpi. (A) Visualized t‐SNE maps of brain cells from TBI and cervical lymphadenectomy+TBI mice at 7 dpi. Maps were based on the expression of 37 different parameters as shown in Table S1. (B) Based on the clusters identified in (A), median intensities for each marker were calculated and plotted as heat maps to identify the respective immune cell populations. (C) Proportion of T cells among total cells in the brain between TBI and cervical lymphadenectomy+TBI mice. (D) The count of T cells per 10^6^ cells in the brain between TBI and cervical lymphadenectomy+TBI mice at 7 dpi. (E) Proportion of CD4^+^ T‐cell 1 (cluster 4) and CD4^+^ T‐cell 2 (cluster 13) among total brain cells at 7 dpi. (F) The count of CD4^+^ T cells per 10^6^ cells in the brain between TBI and cervical lymphadenectomy+TBI mice at 7 dpi. (G) Expression of partial cell markers in CD4^+^ T‐cell 1 and CD4^+^ T‐cell 2. A comprehensive analysis was performed by amalgamating the data obtained from three distinct mice and visually depicting the results using a dumbbell plot. CLNs, cervical lymph nodes; TBI, traumatic brain injury; dpi, day post‐injury.

In this part, we employed CyTOF to investigate the potential impact of cervical lymphadenectomy on the infiltration of CD4^+^ T cells in brain tissue after TBI, as well as the phenotypic characteristics of the CD4^+^ T cells that were affected by this surgical procedure. Through the utilization of cell annotation techniques, which rely on the characterization of specific cell markers, we conducted a comprehensive analysis of T‐cell populations within brain tissue (Figure 2A,B). Our results unveiled a more pronounced perturbation in CD4^+^ T cell abundance relative to CD8^+^ T cells (Figure 2C,D). Moreover, an in‐depth examination of CD4^+^ T cells identified two distinct subsets, CD4^+^ T‐1 (cluster 4) and CD4^+^ T‐2 (cluster 13). The dominant infiltration pattern of the CD4^+^ T‐1 subset decreased after cervical lymphadenectomy (Figure 2E,F). A notable feature of this subset is the significant upregulation of CD11b (Figure 2G).

**FIGURE 3:**
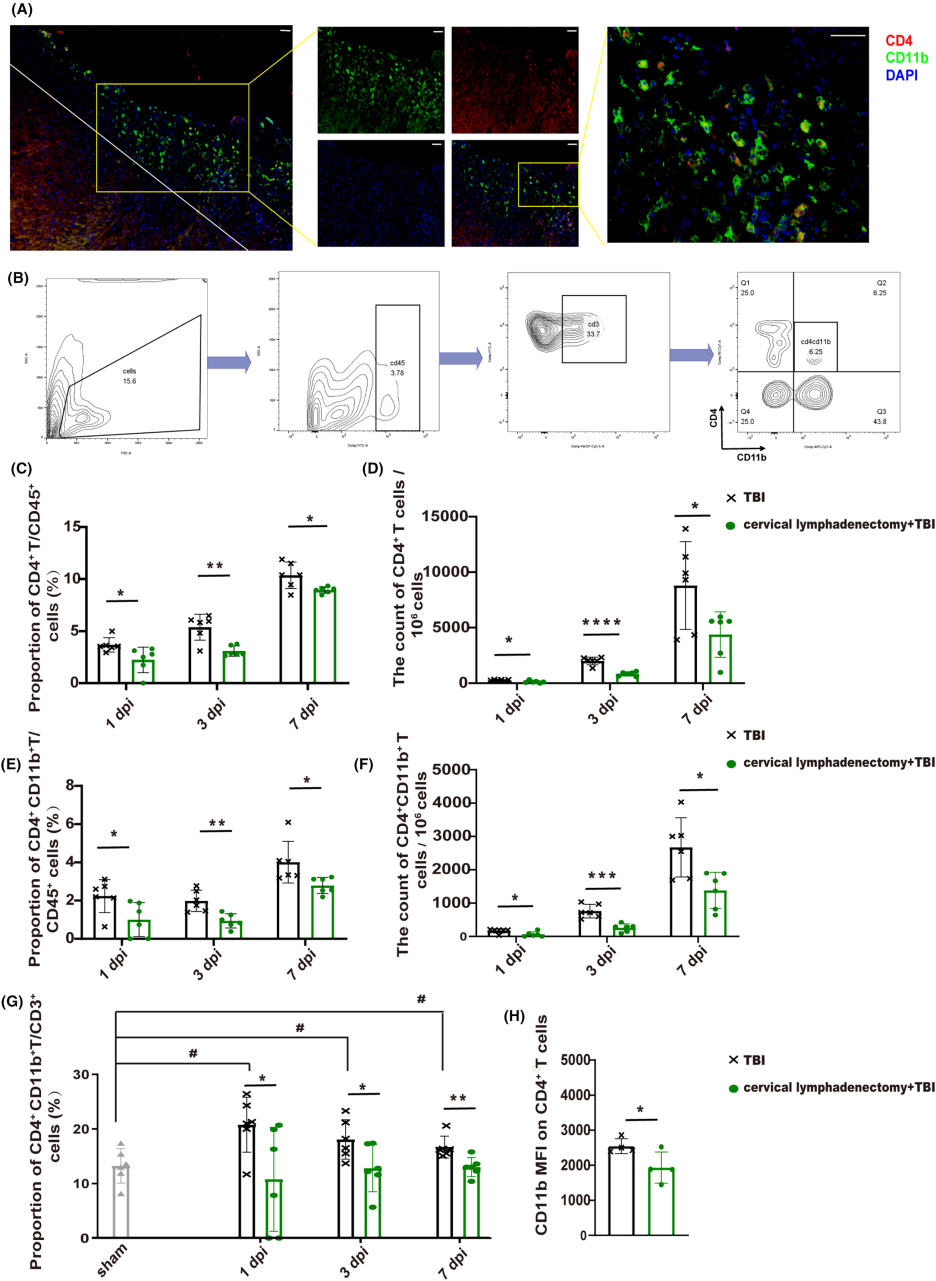
Cervical lymphadenectomy decreased recruitment of brain CD4^+^CD11b^+^ T cells after TBI. (A) Localization of CD4^+^CD11b^+^ T cells in the brain after TBI (red—CD4, green—CD11b, and blue—DAPI). Scale: 100 μm. (B) Logic of cell selection. (C–F) Proportion and count of CD4^+^ T, and CD4^+^ CD11b^+^ T lymphocytes in the injured brain among TBI and cervical lymphadenectomy+TBI mice at 1 dpi, 3 dpi, and 7 dpi (*n* = 6). (G) Proportion of CD4^+^ CD11b^+^ T cells among the CD3^+^ T cells in each group at 1 dpi, 3 dpi, and 7 dpi (*n* = 6). (H) CD11b MFI on CD4^+^ T cells in the injured brain between TBI and cervical lymphadenectomy mice at 7 dpi (*n* = 4). All data are shown as mean ± SD. *p < 0.05, **p < 0.01, ***p < 0.001, and ****p < 0.0001. # *p* < 0.05, ## *p* < 0.01, ### *p* < 0.001, and #### *p* < 0.0001. dpi, day post‐injury; MFI, mean fluorescence intensity; TBI, traumatic brain injury.

The immunofluorescence staining results suggest that the predominant localization of infiltrating CD4^+^CD11b^+^ T cells is within the damaged cortex, aligning with previous research outcomes (Figure 3A).^6^ The proportion and count of infiltrated brain CD4^+^ T cells and CD4^+^CD11b^+^ T cells significantly decreased at 1, 3, and 7 dpi in TBI mice without CLNs, consistent with CyTOF results (Figure 3B–F). Interestingly, although there were still a few CD4^+^CD11b^+^ T cells infiltrated into the brain, the ratio of CD4^+^CD11b^+^ T cells among the CD3^+^ T cells decreased obviously in TBI mice with cervical lymphadenectomy (Figure 3G), and the mean fluorescence intensity (MFI) of CD11b on CD4^+^ T decreased as well (Figure 3H).

### 3.3 Ligating the cervical afferent lymphatic vessels also reduces brain CD4 ^+^ CD11b ^+^ T‐lymphocyte infiltration

**FIGURE 4:**
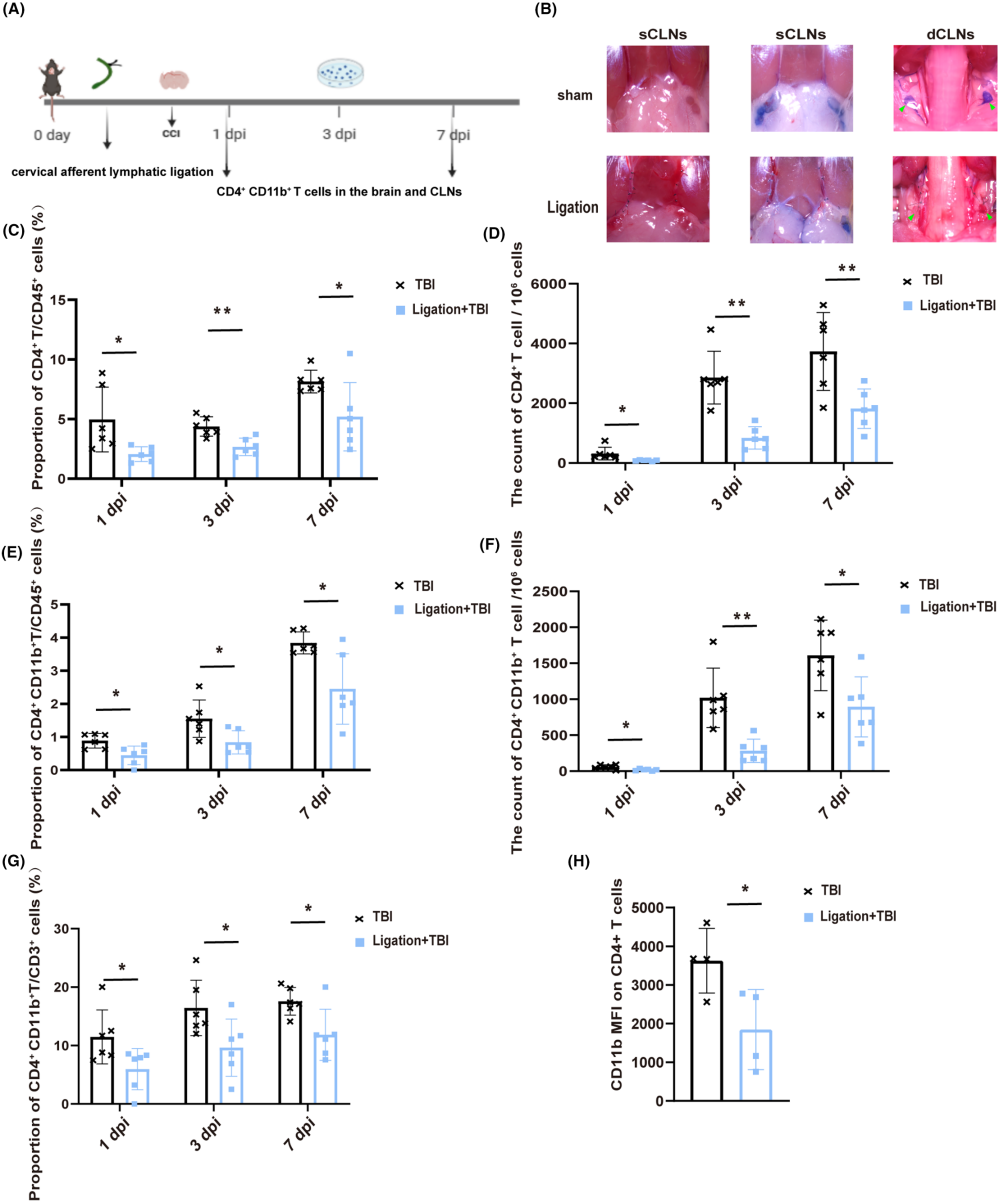
Ligating cervical afferent lymphatic vessels reduces brain T lymphocyte infiltration. (A) The specific experimental procedure for this section. (B) Display drawing of ligating afferent lymphatic vessels of CLNs; dCLNs (green arrow); cervical afferent lymphatic vessels (white arrow). (C–F) Proportion and count of infiltrating CD4^+^ T cells and CD4^+^ CD11b^+^ T cells in the injured brain after TBI at 1 dpi, 3 dpi, and 7 dpi (*n* = 6). (G) Proportion of brain CD4^+^ CD11b^+^ T cells among the CD3^+^ T cells in TBI and Ligation+TBI mice at 1 dpi, 3 dpi, and 7 dpi (*n* = 6). (H) MFI of CD11b on CD4^+^ T cells in the injured brain between TBI and cervical lymphadenectomy mice at 7 dpi (*n* = 4). All data are shown as mean ± SD. *p < 0.05, **p < 0.01, ***p < 0.001, and ****p < 0.0001. dCLNs, deep cervical lymph nodes; dpi, day post‐injury; MFI, mean fluorescence intensity; TBI, traumatic brain injury.

The specific experimental procedure for this section is illustrated in Figure 4A. First, we performed segmental ligation of cervical afferent lymphatic vessels away from the CLNs and then injected a 2% EB solution into the lateral ventricle to evaluate the blocking effect, as shown in Figure 4B. Despite the preservation of CLNs “exit” and CLNs, there was a decrease in the proportion and count of CD4^+^ T and CD4^+^CD11b^+^ T cell in the brain post‐TBI (Figure 4C–F). Additionally, the proportion of CD4^+^CD11b^+^ T cell among CD3^+^ T cell and the MFI of CD11b on CD4^+^ T cell showed similar downward trends (Figure 4G,H).

To determine if this effect is independent of systemic neural activation resulting from the primary TBI, ISF collected from the traumatic brain at 6 h was injected into the lateral ventricle of mice with ligated cervical afferent lymphatic vessels (Figure S1A). The Ligation+ISF group exhibited a reduced infiltration of brain CD4^+^CD11b^+^ T cell compared to the sham+ISF group, confirming our hypothesis (Figure S1B–E). Moreover, the ratio of CD4^+^CD11b^+^ T cell among the CD3^+^ T cell and the MFI of CD11b on CD4^+^ T cell were also decreased (Figure S1F,G).

These findings suggest that the disruption of the brain‐to‐CLN signaling have a directly impacts the recruitment and phenotypic characteristics of infiltrating CD4^+^ T cells into the injured brain.

### 3.4 Blocking brain‐to‐CLN signaling interferes with the release of CD4 ^+^ CD11b ^+^ T cells from CLNs after TBI

**FIGURE 5:**
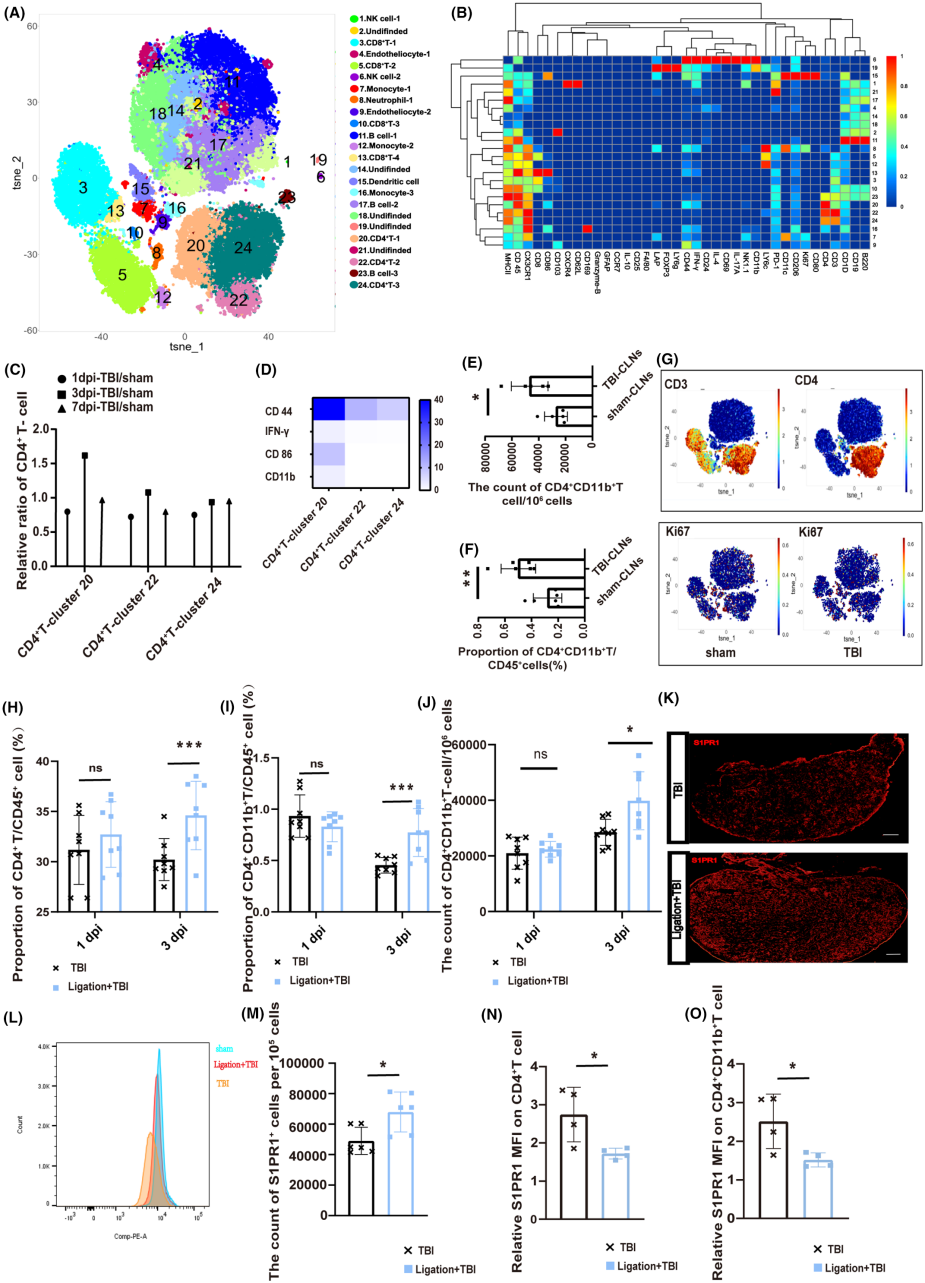
Brain‐to‐CLN signaling promotes the release of CD4^+^CD11b^+^ T cells from CLNs. (A) Visualized t‐SNE maps of CLNs cells from sham and TBI mice at 1 dpi, 3 dpi, and 7 dpi. (B) Heat map was based on the expression of 37 different parameters, as the Table S1 shows. Median intensities for each marker were calculated and plotted as heat maps to identify the respective immune cell populations. (C) The ratio of CLNs CD4^+^ T cells in TBI mice at 1 dpi, 3 dpi, and 7 dpi. Data of CyTOF from three independent mice were pooled together for analysis and visually depicting the results using a dumbbell plot. (D) The relative expression of cell markers (such as CD44, IFN‐γ, CD86, and CD11b) on the CD4^+^ T cells (including clusters 20, 22, and 24). (E and F) The count and proportion of CD4^+^CD11b^+^ T cells in the CLNs at 3 dpi and sham mice. (G) Location of CD3, CD4, and Ki67 in the visualized t‐SNE maps of CLNs between sham and TBI mice. (H and I) The proportion of CD4^+^ T, and CD4^+^CD11b^+^ T cells on the CD45^+^ cells in the CLNs of TBI and Ligation+TBI mice at 1 and 3 dpi (*n* = 8). (J) The count of CD4^+^CD11b^+^ T cells per 10^6^ cells in the CLNs of TBI and Ligation+TBI mice at 1 and 3 dpi (*n* = 8). (K) The CLNs S1PR1 fluorescent staining in TBI and Ligation+TBI mice at 3 dpi. Scale 100 μm. (L and M) The count of S1PR1^+^ cells in the CLNs among TBI and Ligation+TBI mice (*n* = 6). (N and O) The CLNs S1PR1 MFI on CD4^+^ T, CD4^+^CD11b^+^ T cells between TBI and Ligation+TBI mice divided by S1PR1 MFI of sham mice (*n* = 4–6). Data are shown as mean ± SD. *p < 0.05, **p < 0.01, ***p < 0.001, and ****p < 0.0001. CLNs, deep cervical lymph nodes; CyTOF, cytometry by time‐of‐flight analysis; dpi, day post‐injury; IFN‐γ, interferon‐gamma; MFI, mean fluorescence intensity; S1PR1, sphingosine 1‐phosphate receptors‐1; TBI, traumatic brain injury.

Under the undamaged condition, there was a relatively high level of CD4^+^CD11b^+^ T cells in CLNs than in the peripheral blood, indicating the potential for CLNs to assume a distinct role in the infiltration of CD4^+^CD11b^+^ T cells after TBI (Figure S2A,B). CyTOF analysis of CLNs in both sham and TBI mice specifically identified clusters 20, 22, and 24 as CD4^+^ T cells (Figure 5A,B). Although cluster 20 did not exhibit the highest absolute content in the CLNs (Figure S2C), it did display a significant increase in activation and upregulation of functional markers (CD44, IFN‐γ, CD86, and CD11b) at 3 dpi, indicating an intense immune response within the CLNs (Figure 5C,D). Consistent outcomes were obtained through subsequent FACS validation of CD4^+^CD11b^+^ T cells at 3 dpi (Figure 5E,F). This observation suggests a temporal delay in TBI signals by CLNs, which may partially explain the peak infiltration of CD4^+^ T cells into brain tissue occurring at 7 dpi.

Ki67, a cell proliferation marker, was mainly detected in monocyte or dendritic cells and had lower expression in T cells (Figure 5G). This finding imply that the observed outcomes might be attributed to the activation of brain‐to‐CLNs signaling during the acute stage post‐TBI, consequently facilitating the release of CD4^+^ T cells.

We disrupted brain‐to‐CLN signaling by ligating cervical afferent lymphatic vessels to test our hypothesis. The proportion of CD4^+^ T cells and CD4^+^CD11b^+^ T cells in CLNs significantly increased compared to TBI mice at 3 dpi (Figure 5H,I), and the count of CD4^+^CD11b^+^ T cells increased either (Figure 5J). The overall expression of sphingosine‐1‐phosphate receptor‐1 (S1PR1) increased, a marker for T‐cell release (Figure 5K–M). This is consistent with the increased proportion of CD4^+^ T cells in the CLNs. However, the relative MFI of S1PR1 on CD4^+^ T and CD4^+^CD11b^+^ T cells, representing their “escape capacity”, decreased at 3 dpi (Figure 5N,O). However, a reverse trend was noted at 7 dpi (Figure S2D), potentially due to the recanalization of the previously ligated lymphatic vessels. Injection of a fluorescent tracer confirmed lymphatic recanalization (Figure S2E–G), providing further support for the role of brain‐to‐CLNs signaling in promoting the release of CD4^+^ T cells from the CLNs.

In light of the potential confounding effects of lymphatic channel recanalization following CLNs ligation, CLNs excision was utilized as an intervention tactic to evaluate the levels of CD4^+^CD11b^+^ T cells in peripheral blood. There was a decreased abundance of peripheral blood CD4^+^CD11b^+^ T cells on the seventh day post‐CLNs excision relative to those in the TBI group simply (Figure S2H,I). This provided additional evidence to corroborate our point.

Similarly, to eliminate the potential impact of systemic neural activation caused by the primary TBI, ISF was injected into the lateral ventricle of mice whose cervical afferent lymphatic vessels have been ligated. Then, the ligated CLNs were detected through FACS. In the Ligation+Ag mice, there was an increase in the proportion of CD4^+^ T lymphocytes, accompanied by a decrease in the MFI of S1PR1 on the surface of lymphocytes compared to the sham+Ag mice (Figure S2J–M).

These findings indicate that TBI induces the generation of injury‐related signals that enhance the release of CD4^+^CD11b^+^ T cells in CLNs.

### 3.5 CD4 ^+^ CD11b ^+^ T lymphocyte aggravating brain injury after TBI

**FIGURE 6:**
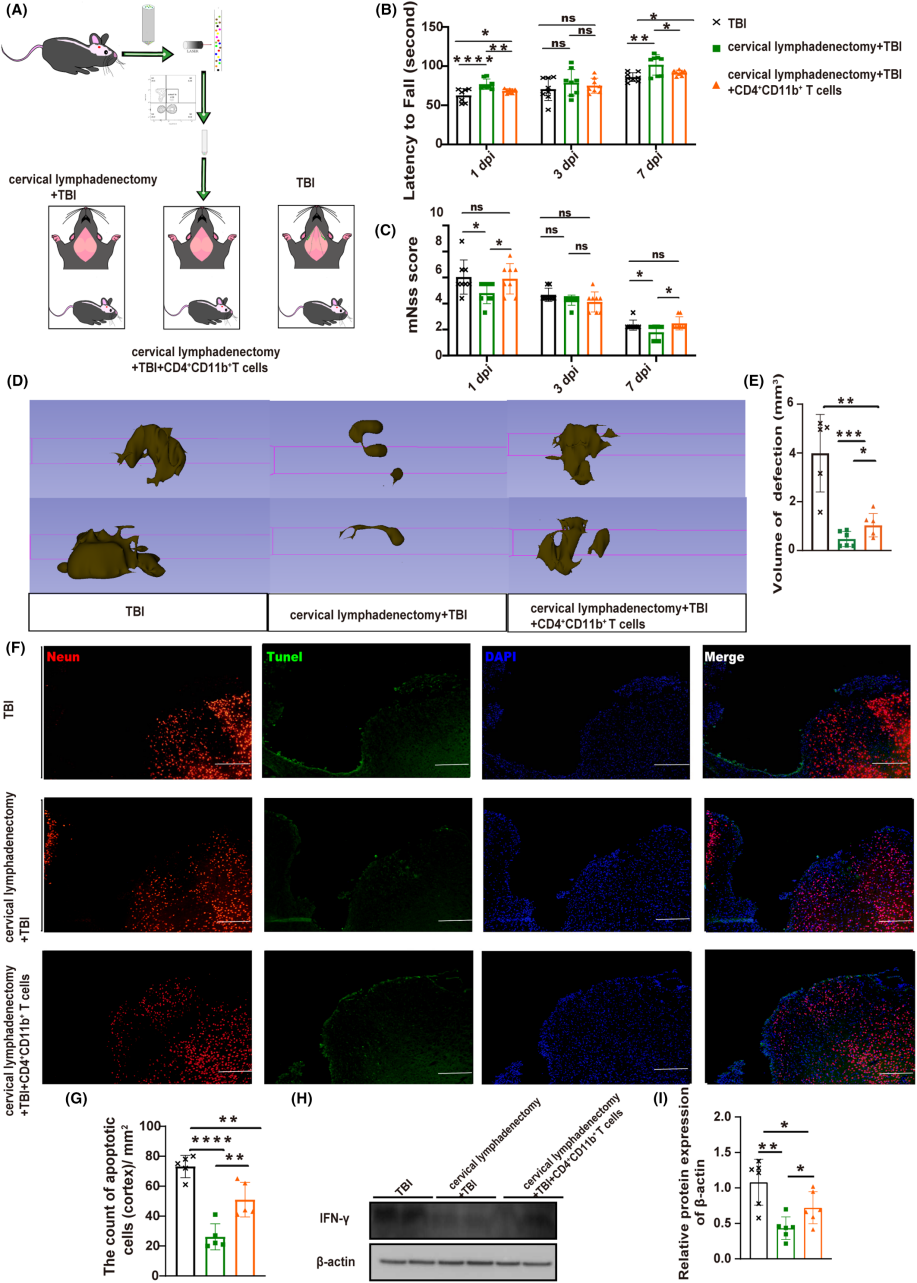
CD4^+^CD11b^+^ T cells aggravated brain injury. (A) Schematic diagram of experimental operation. (B) The statistics of the rotarod experiment in each group (*n* = 8). (C) mNss results (*n* = 8). (D and E) The defect lesions of mice with 3D Slicer and the statistical volumetric histogram of defect lesions (*n* = 5–6). (F) Fluorescent staining of apoptosis. Fluorescence color: NeuN, red channel; TUNEL, green channel; DAPI, blue channel. Scale 100 μm. (G) The total number of neuro apoptosis on both sides of the lesion (*n* = 5). The area of measurement for statistical purposes is approximately 1 mm^2^ on each side of the lesion. (H) Representative western blots of β‐actin and IFN‐γ. (I) Quantification of relative protein expression normalized to the optical density of β‐actin (*n* = 6). All data are shown as mean ± SD. *p < 0.05, **p < 0.01, ***p < 0.001, and ****p < 0.0001. DAPI, 4′,6‐diamidino‐2‐phenylindole; dpi, day post‐injury; mNSS, modified neurological severity score; MRI, Magnetic Resonance Imaging; TUNEL, terminal deoxynucleotidyl transferase‐mediated dUTP nick end labeling (TUNEL) staining.

To gain further insights into the functional implications of CD4^+^CD11b^+^ T cells in TBI, we investigated whether replenishing this specific subset would exacerbate the pathogenesis of TBI. To investigate the impact of CD4^+^CD11b^+^ T‐cell supplementation on TBI, we performed the following experiment (Figure 6A): Isolated CD4^+^CD11b^+^ T cells from the CLNs at 3 dpi were injected into the lateral ventricles of mice with surgically removed CLNs, while an equal volume of saline was injected into two separate control groups. Motor function was assessed in all groups at 1, 3, and 7 dpi, and mice receiving supplemental CD4^+^CD11b^+^ T cells showed some functional impairments compared to TBI mice without CLNs (Figure 6B,C). However, these impairments were not sufficient to compensate for the deficiencies caused by the presence of CLNs. Brain edema at 7 dpi was evaluated using MRI, revealing more pronounced edema in the group receiving CD4^+^CD11b^+^ T‐cell supplementation (Figure S3A,B). Using 3D Slicer software, lesion volume reconstruction revealed that TBI mice exhibited enormous brain defect volume, followed by CD4^+^CD11b^+^ T‐cell‐supplemented mice, aligning with functional assessment outcomes (Figure 6D,E). Statistical analysis of apoptosis on both sides of the lesion produced consistent results (Figure 6F,G). T‐cell‐associated inflammatory cytokine IFN‐γ detection findings demonstrate congruity (Figure 6H,I).

These findings emphasize the role of CD4^+^CD11b^+^ T cells derived from the CLNs in exacerbating the pathologic consequences of TBI. However, it is noteworthy that the harmful effect attributed solely to these cells is insufficient to fully understand the impact of CLNs on TBI.

## 4 DISCUSSION

Our study provides evidence supporting the role of brain‐to‐CLN signaling in CD4^+^ T‐cells infiltration into the traumatic brain and describes the phenotype of CD4^+^ T cells influenced by this signaling. We found that removing CLNs or blocking this signaling reduces the infiltration of CD4^+^ T cells, especially CD4^+^CD11b^+^ T cells. Supplementing CD4^+^CD11b^+^ T cells from CLNs worsened acute brain injury post‐TBI. These findings highlight the importance of brain‐to‐CLN signaling in regulating the neuroimmunity.

Although proposed in 2019, research on brain‐to‐CLN signaling predates this concept. The term “brain‐derived antigens” has been used in EAE and stroke research for many years,^27^ often represented by injury‐related proteins like myelin basic protein and oligodendrocyte glycoprotein,^28^ but it fails to encompass the diverse debris and molecules post‐TBI. By adopting the concept of brain‐to‐CLN signaling, Lo and Hayakawa found that removing sCLNs significantly reduced macrophage infiltration and mitigated neural damage after stroke.^13^ Consensus is lacking on defining this signaling, but researchers are increasingly interested in its neuroimmune implications. Intervening in brain‐to‐CLN signaling may become a novel treatment trend for related conditions. Previous research has primarily focused on the function of CLNs in EAE mice,^17^, ^29^ with limited investigations specifically targeting TBI. Our findings fill a critical gap by revealing the role of CLNs in modulating neuroimmunity and identifying the detrimental role of specific CD4^+^CD11b^+^ T cells post‐TBI. However, based on the current research findings, the role of other peripheral immune organs in T‐cell infiltration cannot be ignored.

CD11b is an essential adhesion molecule that plays a crucial role in mediating the inflammatory response and shows potential involvement in transendothelial migration of T cells following their activation.^30^ CD11b expression on T cells is crucial for the differentiation of pathogenic T cells, and its deficiency results in delayed and attenuated EAE.^31^, ^32^, ^33^, ^34^ CD11b^+^ T cells exhibit an activated effector phenotype and their presence suggests a contribution to neuroinflammation. While we cannot confirm its exclusive presence in CLNs, the proportion of CD11b^+^ T cells in CLNs is higher than in the blood, suggesting a strong association. It is noteworthy that although the ISF post‐TBI has been demonstrated to enhance the expression of CD4^+^CD11b T cells in CLNs, it is probable that other factors also contribute to this phenomenon. Expression of CD4^+^CD11b^+^ T cells in CLNs was increased in both the TBI and Ligation+TBI groups at 1 dpi, whereas sham mice showed lower levels of expression. These observations suggest that injury‐induced systemic neural activation may also contribute to the upregulation of CD11b. It appears that the signaling of injury‐derived products following TBI primarily controls the release of CD4^+^CD11b^+^ T cells from CLNs. More research is needed to understand the precise role of this T cell in TBI mice.

It is important to clarify that our research primarily aims to emphasize the involvement of brain‐to‐CLN signaling in the regulation of CD4^+^ T‐cell brain infiltration and its associated phenotypes post‐TBI, rather than elucidating the exclusive causal relationship between brain‐to‐CLN signaling blockade and the reduction of brain injury. Bolte's results suggested that pre‐existing impairment of mLVs function exacerbates brain damage after TBI.^35^ However, it is important to note that their methods altered the immune environment of the meninges to varying degrees, providing a modified baseline state. The pathologic progression of TBI is influenced by multiple factors, including the site of injury, hematoma drainage rate, and activation of the peripheral immune system. The intricacy of the mechanisms involved in TBI hinders the attribution of its effects to a single factor, which may account for the diverse functional outcomes observed in our studies. While Bolte's study did not specifically focus on T cells, the findings of Louveau et al. align with our result, demonstrating that ablation of mLVs restricts infiltration of CD4^+^ T cells into the brain and improves prognosis in EAE mice.^36^

Drawing upon previous studies and our own findings, we sought to describe the CD4^+^ T‐cell infiltration pattern associated with CLNs post‐TBI. As brain injury‐related products accumulate, they are drained through the mLVs to CLNs, where antigen‐presenting cells process these signals. CD4^+^ T cells enter CLNs through high endothelial venules and interact with antigen‐presenting cells.^37^, ^38^, ^39^ It is highly probable that this process of recognition and processing takes place within a time frame of approximately 1–3 days after TBI, thereby facilitating the capacity of CD4^+^ T cells to be released from CLNs and infiltrate the injured brain. The interaction between S1PR1 and sphingosine 1‐phosphate (S1P) plays a crucial role, as it leads to the internalization of S1PR1 and creates a gradient that prompts CD4^+^ T cells to escape from CLNs and infiltrate the brain by crossing the compromised BBB.^40^, ^41^, ^42^, ^43^ Therefore, removing CLNs or blocking brain‐to‐CLN signaling prevents CD4^+^ T cells from escaping CLNs and infiltrating the brain.

Our research has limitations including identifying the precise components of brain‐to‐CLN signaling, yet understanding these constituents is crucial for targeted therapeutic advancements. The CyTOF analysis showed that cervical lymphadenectomy impacts various cell types, including CD4^+^ T cells, microglia, macrophages, NK cells, and dendritic cells, indicating that factors beyond CD4^+^CD11b^+^ T cells may also contribute to the observed reduction in brain damage. Further research is needed to understand the impact of this signaling on brain atlases. Furthermore, the CyTOF data in this study comes from a sample of three independent mice, which may lead to potential deviations. Additional visual evidence is needed to confirm CLNs as a primary source of CD4^+^CD11b^+^ T cells. Future research aims to address these unresolved questions.

## 5 CONCLUSIONS

Our study investigates the impact of brain‐to‐CLN signaling on the release of CD4^+^CD11b^+^ T cells from CLNs, which contributes to their infiltration into the brain and the subsequent neuroimmune and neuroinflammatory responses in the acute phase of TBI. Modulating this signal holds the potential to offer novel therapeutic approaches for managing TBI.

## AUTHOR CONTRIBUTIONS

WWJ, XHL, and CG made significant contributions to the literature collection, study design, data interpretation, and article writing. RCJ, JNZ, MQL, JYJ, and JHH conducted article reviews, revisions, and quality assessments. WWJ, YPC, and MN conducted FACS and CyTOF analyses. DW and YBF conducted PCR and MRI procedures. SYD, TL, YQ, CRW, and WWJ contributed to data interpretation and study oversight. All authors carefully reviewed and approved the final article.

## FUNDING INFORMATION

This work was supported by grants from the National Natural Science Foundation of China (grant 82071390 and 82271394 to R.J., 82001323 to C.G.).

## CONFLICT OF INTEREST STATEMENT

The authors declare that they have no competing interests.

## Supporting information


File S1

Figure S1

Figure S2

Figure S3

Table S1

## Data Availability

All data generated and analyzed during this study are included in this published article and its File S1.

## References

[1] Corrigan F , Mander KA , Leonard AV , Vink R . Neurogenic inflammation after traumatic brain injury and its potentiation of classical inflammation. J Neuroinflammation. 2016;13(1):264. doi:10.1186/s12974-016-0738-9 27724914 PMC5057243

[2] Collaborators GBDN . Global, regional, and national burden of neurological disorders, 1990‐2016: a systematic analysis for the global burden of disease study 2016. Lancet Neurol. 2019;18(5):459‐480. doi:10.1016/S1474-4422(18)30499-X 30879893 PMC6459001

[3] Corps KN , Roth TL , McGavern DB . Inflammation and neuroprotection in traumatic brain injury. JAMA Neurol. 2015;72(3):355‐362. doi:10.1001/jamaneurol.2014.3558 25599342 PMC5001842

[4] Dinet V , Petry KG , Badaut J . Brain‐immune interactions and Neuroinflammation after traumatic brain injury. Front Neurosci. 2019;13:1178. doi:10.3389/fnins.2019.01178 31780883 PMC6861304

[5] Bao W , Lin Y , Chen Z . The peripheral immune system and traumatic brain injury: insight into the role of T‐helper cells. Int J Med Sci. 2021;18(16):3644‐3651. doi:10.7150/ijms.46834 34790036 PMC8579286

[6] Fee D , Crumbaugh A , Jacques T , et al. Activated/effector CD4+ T cells exacerbate acute damage in the central nervous system following traumatic injury. J Neuroimmunol. 2003;136(1–2):54‐66. doi:10.1016/s0165-5728(03)00008-0 12620643

[7] Jonathan Kipnis EY , Porat Z , Cohen A , et al. T cell immunity to copolymer 1 confers neuroprotection on the damaged optic nerve: possible therapy for optic neuropathies. Proc Natl Acad Sci USA. 2000;97(13):7446‐7451. 10861010 10.1073/pnas.97.13.7446 PMC16565

[8] Harald H , Hofstettera DLS , Liub F , et al. Autoreactive T cells promote post‐traumatic healing in the central nervous system. J Neuroimmunol. 2003;134:25‐34. 12507769 10.1016/s0165-5728(02)00358-2

[9] Ellwardt E , Walsh JT , Kipnis J , Zipp F . Understanding the role of T cells in CNS homeostasis. Trends Immunol. 2016;37(2):154‐165. doi:10.1016/j.it.2015.12.008 26775912

[10] Engelhardt B , Ransohoff RM . The ins and outs of T‐lymphocyte trafficking to the CNS: anatomical sites and molecular mechanisms. Trends Immunol. 2005;26(9):485‐495. doi:10.1016/j.it.2005.07.004 16039904

[11] Aspelund A , Antila S , Proulx ST , et al. A dural lymphatic vascular system that drains brain interstitial fluid and macromolecules. J Exp Med. 2015;212(7):991‐999. doi:10.1084/jem.20142290 26077718 PMC4493418

[12] Yamada S . Cerebrospinal fluid dynamics. Croat Med J. 2021;62(4):399‐410. doi:10.3325/cmj.2021.62.399 34472743 PMC8491047

[13] Esposito E , Ahn BJ , Shi J , et al. Brain‐to‐cervical lymph node signaling after stroke. Nat Commun. 2019;10(1):5306. doi:10.1038/s41467-019-13324-w 31757960 PMC6876639

[14] Bartholomaus I , Kawakami N , Odoardi F , et al. Effector T cell interactions with meningeal vascular structures in nascent autoimmune CNS lesions. Nature. 2009;462(7269):94‐98. doi:10.1038/nature08478 19829296

[15] Bolte AC , Lukens JR . Neuroimmune cleanup crews in brain injury. Trends Immunol. 2021;42(6):480‐494. doi:10.1016/j.it.2021.04.003 33941486 PMC8165004

[16] van Zwam M , Huizinga R , Heijmans N , et al. Surgical excision of CNS‐draining lymph nodes reduces relapse severity in chronic‐relapsing experimental autoimmune encephalomyelitis. J Pathol. 2009;217(4):543‐551. doi:10.1002/path.2476 19023878

[17] Harling‐Berg CJ , Park TJ , Knopf PM . Role of the cervical lymphatics in the Th2‐type hierarchy of CNS immune regulation. J Neuroimmunol. 1999;101(2):111‐127. doi:10.1016/s0165-5728(99)00130-7 10580795

[18] Mackay CR , Kimpton WG , Brandon MR , Cahill RN . Lymphocyte subsets show marked differences in their distribution between blood and the afferent and efferent lymph of peripheral lymph nodes. J Exp Med. 1988;167(6):1755‐1765. 3290379 10.1084/jem.167.6.1755 PMC2189690

[19] Wu H , Zheng J , Xu S , et al. Mer regulates microglial/macrophage M1/M2 polarization and alleviates neuroinflammation following traumatic brain injury. J Neuroinflammation. 2021;18(1):2. doi:10.1186/s12974-020-02041-7 33402181 PMC7787000

[20] Gao C , Qian Y , Huang J , et al. A three‐day consecutive fingolimod administration improves neurological functions and modulates multiple immune responses of CCI mice. Mol Neurobiol. 2017;54(10):8348‐8360. doi:10.1007/s12035-016-0318-0 27924525

[21] Kataru RP , Kim H , Jang C , et al. T lymphocytes negatively regulate lymph node lymphatic vessel formation. Immunity. 2011;34(1):96‐107. doi:10.1016/j.immuni.2010.12.016 21256057

[22] Mitsialis V , Wall S , Liu P , et al. Single‐cell analyses of colon and Blood reveal distinct immune cell signatures of ulcerative Colitis and Crohn's disease. Gastroenterology. 2020;159(2):591‐608 e10. doi:10.1053/j.gastro.2020.04.074 32428507 PMC8166295

[23] Lv C , Han S , Sha Z , et al. Cerebral glucagon‐like peptide‐1 receptor activation alleviates traumatic brain injury by glymphatic system regulation in mice. CNS Neurosci Ther. 2023;29(12):3876‐3888. doi:10.1111/cns.14308 37353947 PMC10651945

[24] Chen J , Wang X , Hu J , et al. FGF20 protected against BBB disruption after traumatic brain injury by upregulating junction protein expression and inhibiting the inflammatory response. Front Pharmacol. 2020;11:590669. doi:10.3389/fphar.2020.590669 33568994 PMC7868342

[25] Jassam YN , Izzy S , Whalen M , McGavern DB , El Khoury J . Neuroimmunology of traumatic brain injury: time for a paradigm shift. Neuron. 2017;95(6):1246‐1265. doi:10.1016/j.neuron.2017.07.010 28910616 PMC5678753

[26] Bai R , Gao H , Han Z , et al. Flow cytometric characterization of T cell subsets and microglia after repetitive mild traumatic brain injury in rats. Neurochem Res. 2017;42(10):2892‐2901. doi:10.1007/s11064-017-2310-0 28620825 PMC5626793

[27] Planas AM , Gomez‐Choco M , Urra X , Gorina R , Caballero M , Chamorro A . Brain‐derived antigens in lymphoid tissue of patients with acute stroke. J Immunol. 2012;188(5):2156‐2163. doi:10.4049/jimmunol.1102289 22287710

[28] de Vos AF , van Meurs M , Brok HP , et al. Transfer of central nervous system autoantigens and presentation in secondary lymphoid organs. J Immunol. 2002;169(10):5415‐5423. doi:10.4049/jimmunol.169.10.5415 12421916

[29] Phillips MJ , Needham M , Weller RO . Role of cervical lymph nodes in autoimmune encephalomyelitis in the Lewis rat. J Pathol. 1997;182(4):457‐464. doi:10.1002/(SICI)1096-9896(199708)182:4<457::AID-PATH870>3.0.CO;2-Y 9306968

[30] von Asmuth EJ , van der Linden CJ , Leeuwenberg JF , Buurman WA . Involvement of the CD11b/CD18 integrin, but not of the endothelial cell adhesion molecules ELAM‐1 and ICAM‐1 in tumor necrosis factor‐alpha‐induced neutrophil toxicity. J Immunol. 1991;147(11):3869‐3875. 1719092

[31] Muto S , Vĕtvicka V , Ross GD . CR3 (CD11b/CD18) expressed by cytotoxic T cells and natural killer cells is upregulated in a manner similar to neutrophil CR3 following stimulation with various activating agents. J Clin Immunol. 1993;13(3):175‐184. 8100571 10.1007/BF00919970

[32] Wagner C , Hansch GM , Stegmaier S , Denefleh B , Hug F , Schoels M . The complement receptor 3, CR3 (CD11b/CD18), on T lymphocytes: activation‐dependent up‐regulation and regulatory function. Eur J Immunol. 2001;31(4):1173‐1180. doi:10.1002/1521-4141(200104)31:4<1173::aid-immu1173>3.0.co;2-9 11298342

[33] Wu H , Rodgers JR , Perrard XY , et al. Deficiency of CD11b or CD11d results in reduced staphylococcal enterotoxin‐induced T cell response and T cell phenotypic changes. J Immunol. 2004;173(1):297‐306. doi:10.4049/jimmunol.173.1.297 15210787

[34] Bullard DC , Hu X , Schoeb TR , Axtell RC , Raman C , Barnum SR . Critical requirement of CD11b (Mac‐1) on T cells and accessory cells for development of experimental autoimmune encephalomyelitis. J Immunol. 2005;175(10):6327‐6333. doi:10.4049/jimmunol.175.10.6327 16272284

[35] Bolte AC , Dutta AB , Hurt ME , et al. Meningeal lymphatic dysfunction exacerbates traumatic brain injury pathogenesis. Nat Commun. 2020;11(1):4524. doi:10.1038/s41467-020-18113-4 32913280 PMC7483525

[36] Louveau A , Herz J , Alme MN , et al. CNS lymphatic drainage and neuroinflammation are regulated by meningeal lymphatic vasculature. Nat Neurosci. 2018;21(10):1380‐1391. doi:10.1038/s41593-018-0227-9 30224810 PMC6214619

[37] De Virgiliis F , Oliva VM , Kizil B , Scheiermann C . Control of lymph node activity by direct local innervation. Trends Neurosci. 2022;45(9):704‐712. doi:10.1016/j.tins.2022.06.006 35820971

[38] Baeyens A , Bracero S , Chaluvadi VS , Khodadadi‐Jamayran A , Cammer M , Schwab SR . Monocyte‐derived S1P in the lymph node regulates immune responses. Nature. 2021;592(7853):290‐295. doi:10.1038/s41586-021-03227-6 33658712 PMC8475585

[39] Hunter MC , Teijeira A , Halin C . T cell trafficking through lymphatic vessels. Front Immunol. 2016;7:613. doi:10.3389/fimmu.2016.00613 28066423 PMC5174098

[40] Schwab SR , Pereira JP , Matloubian M , Xu Y , Huang Y , Cyster JG . Lymphocyte sequestration through S1P lyase inhibition and disruption of S1P gradients. Science. 2005;309(5741):1735‐1739. 16151014 10.1126/science.1113640

[41] Liu CH , Thangada S , Lee MJ , Van Brocklyn JR , Spiegel S , Hla T . Ligand‐induced trafficking of the sphingosine‐1‐phosphate receptor EDG‐1. Mol Biol Cell. 1999;10(4):1179‐1190. doi:10.1091/mbc.10.4.1179 10198065 PMC25247

[42] Lo CG , Xu Y , Proia RL , Cyster JG . Cyclical modulation of sphingosine‐1‐phosphate receptor 1 surface expression during lymphocyte recirculation and relationship to lymphoid organ transit. J Exp Med. 2005;201(2):291‐301. doi:10.1084/jem.20041509 15657295 PMC2212802

[43] Yanagida K , Hla T . Vascular and Immunobiology of the circulatory sphingosine 1‐phosphate gradient. Annu Rev Physiol. 2017;79:67‐91. doi:10.1146/annurev-physiol-021014-071635 27813829 PMC5500220

