# Supplementary material for: CD4 + CD11b + T cells infiltrate and aggravate the traumatic brain injury depending on brain‐to‐cervical lymph node signaling
Source: CNS Neurosci Ther. 2024 Mar 11;30(3):e14673. doi: 10.1111/cns.14673 (PMC10928342; doi:10.1111/cns.14673)
Supplement: Supplementary file 1 — File S1 [file CNS-30-e14673-s005.pdf]

Full unedited blot for Figure 1F

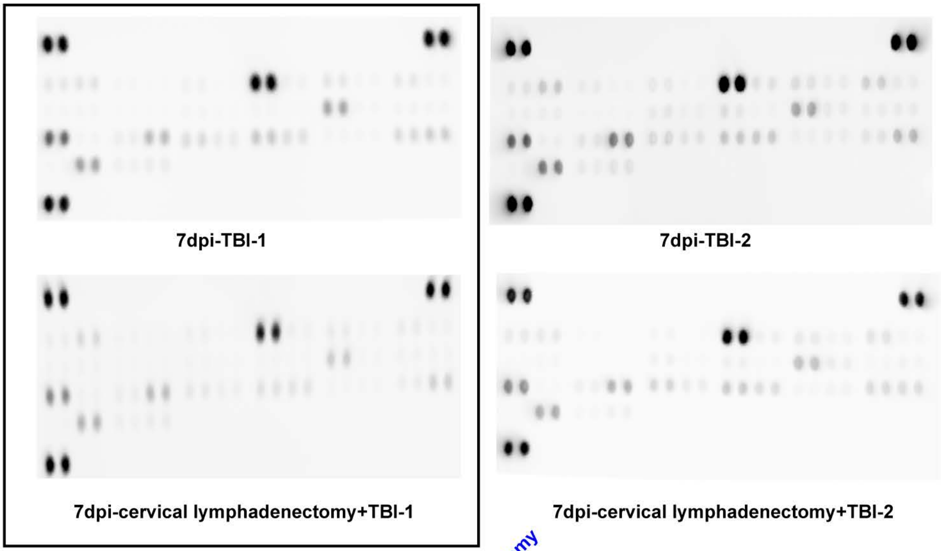

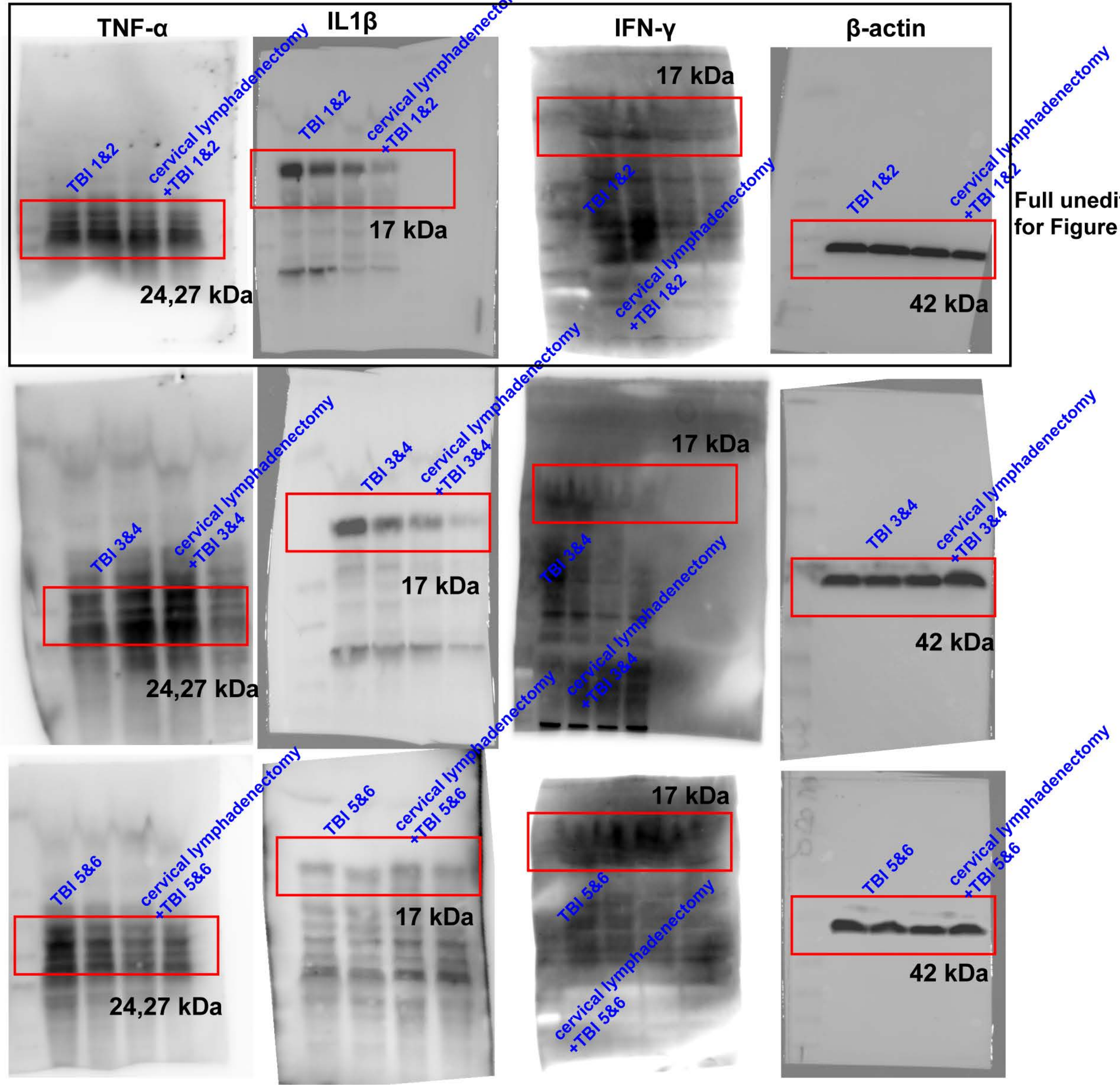

Full unedited blot for Figure 1H

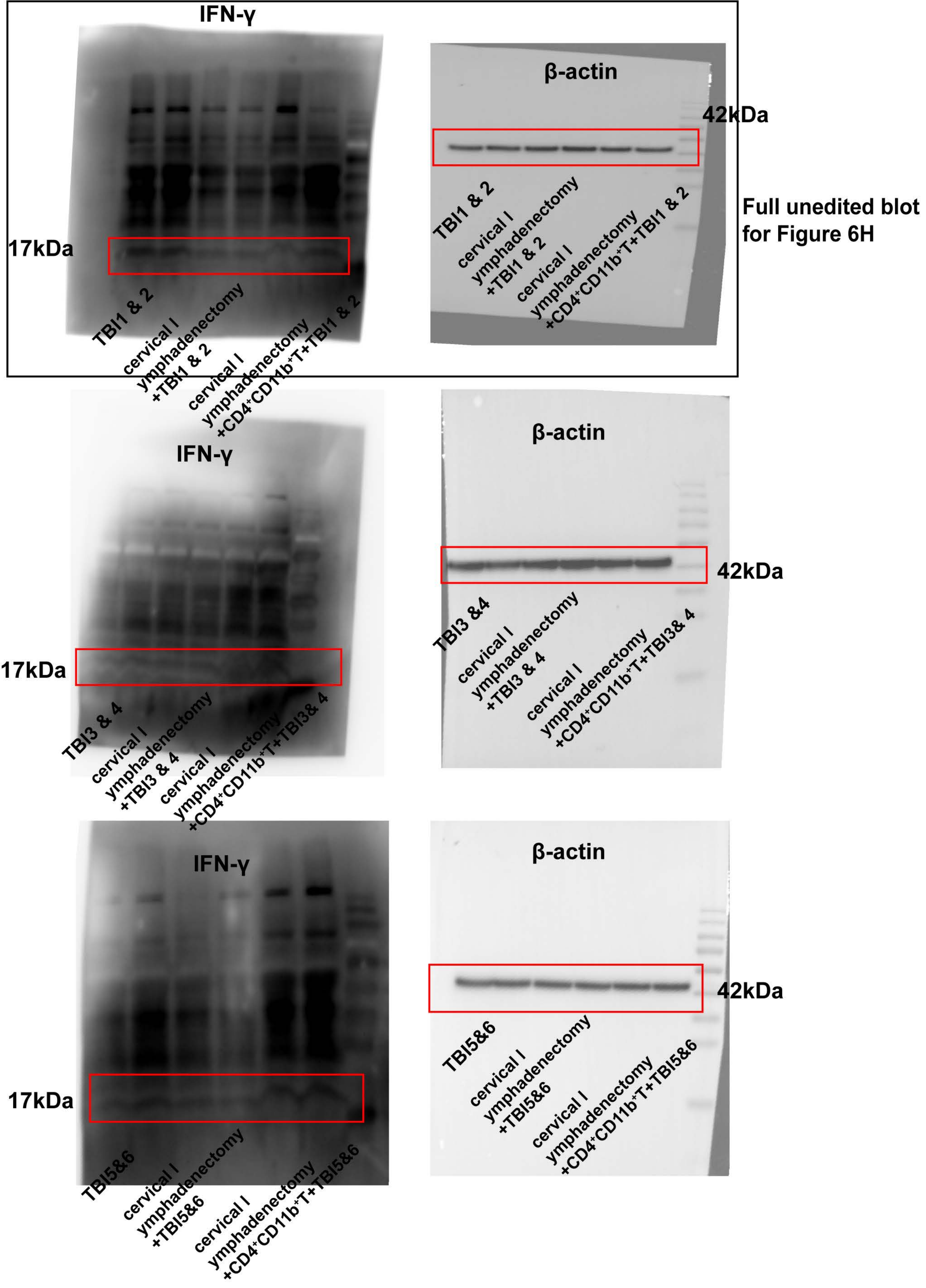

Full unedited blot for Figure 6H
