# Supplementary figures and images for: CD4 + CD11b + T cells infiltrate and aggravate the traumatic brain injury depending on brain‐to‐cervical lymph node signaling
Source: CNS Neurosci Ther. 2024 Mar 11;30(3):e14673. doi: 10.1111/cns.14673 (PMC10928342; doi:10.1111/cns.14673)

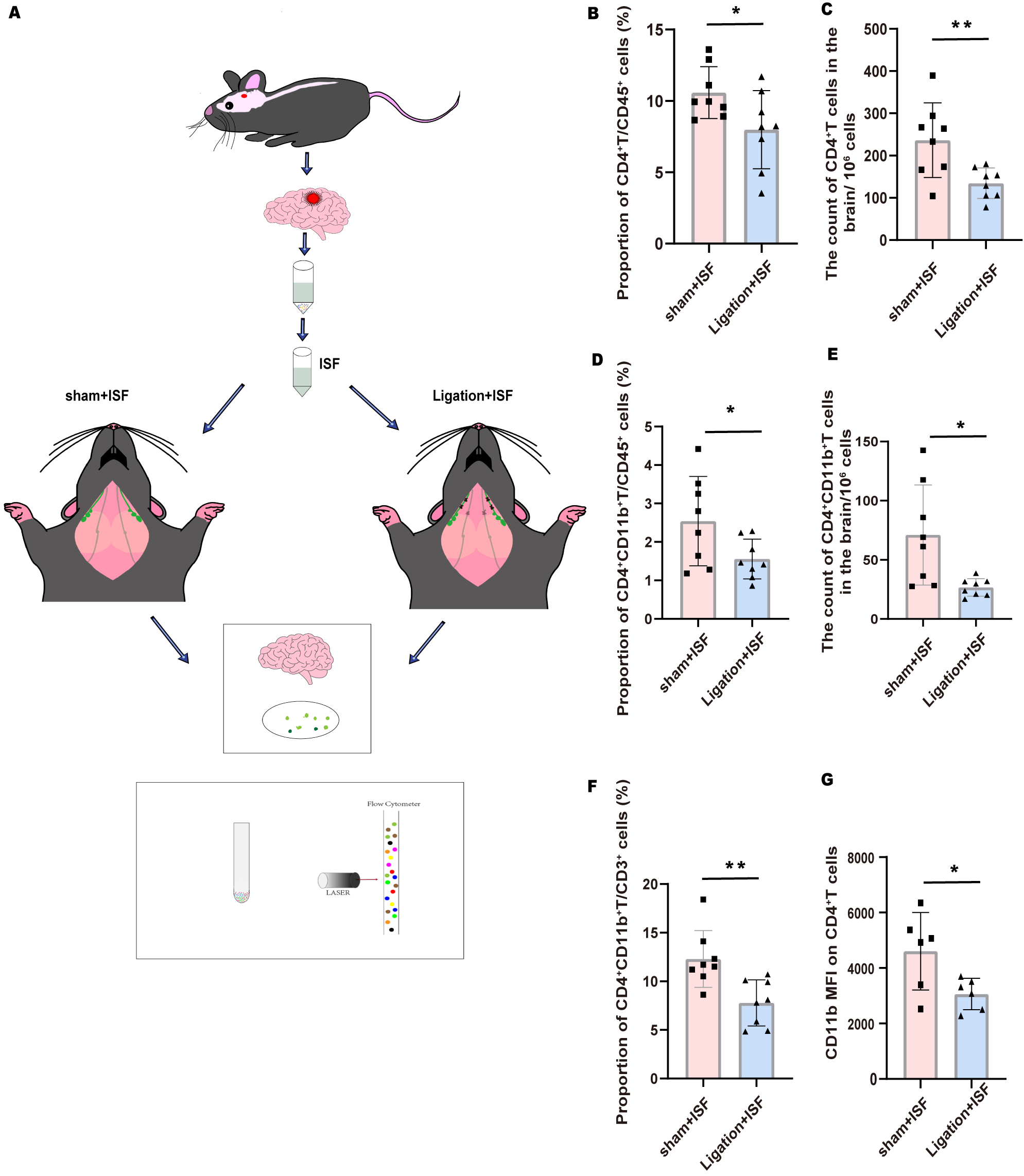

Supplement: Supplementary file 2 — Figure S1 [file CNS-30-e14673-s001.tif]

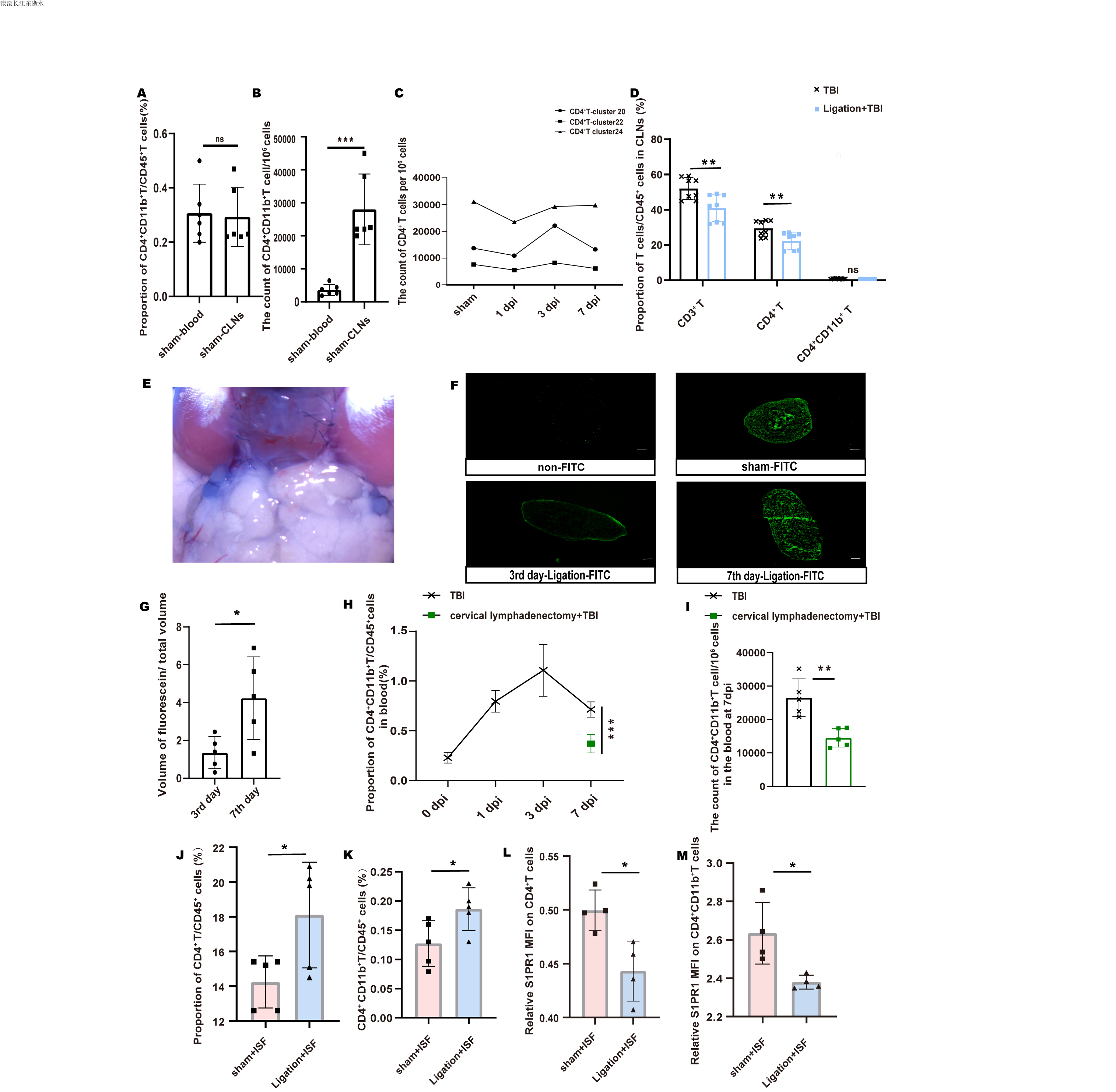

Supplement: Supplementary file 3 — Figure S2 [file CNS-30-e14673-s002.tif]

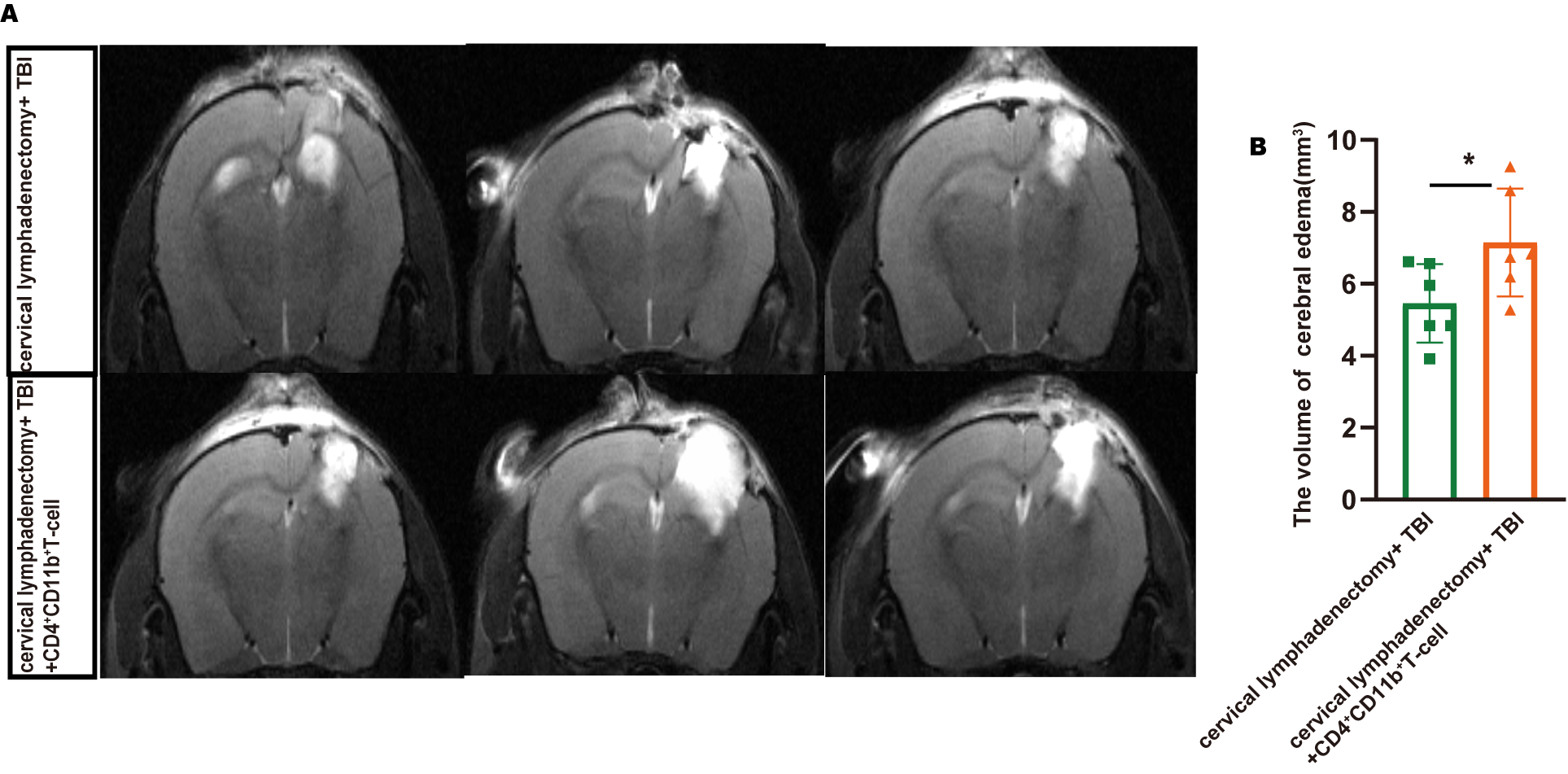

Supplement: Supplementary file 4 — Figure S3 [file CNS-30-e14673-s003.tif]
