# Supplementary material for: CD4 + CD11b + T cells infiltrate and aggravate the traumatic brain injury depending on brain‐to‐cervical lymph node signaling
Source: CNS Neurosci Ther. 2024 Mar 11;30(3):e14673. doi: 10.1111/cns.14673 (PMC10928342; doi:10.1111/cns.14673)
Supplement: Supplementary file 5 — Table S1 [file CNS-30-e14673-s004.doc]

Table S1

Reagents & Antibodies

| Product | Producer | Code |
| --- | --- | --- |
| CD45 | Fluidigm | 3089005B |
| CD103 | R＆D | AF1990 |
| PD-1 | Abcam | ab95789 |
| NK1.1 | Abcam | ab289553 |
| CD44 | Abcam | ab112178 |
| IFN-γ | R＆D | MAB485 |
| CD69 | Abcam | ab25190 |
| CD206 | R＆D | AF2535 |
| CD25 | Biolegend | 101902 |
| CD4 | Abcam | ab251480 |
| CD11b | Fluidigm | 3149028D |
| CD24 | Fluidigm | 3150009B |
| IL17a | R＆D | MAB7211 |
| CD3 | Fluidigm | 3152004B |
| LAP | R＆D | MAB7666 |
| CD8 | R＆D | MAB116 |
| F4/80 | Abcam | ab16911 |
| CD19 | Abcam | ab240533 |
| IL10 | Fluidigm | 3158002B |
| CXCR4 | Fluidigm | 3159030B |
| CD62 | Fluidigm | 3160008B |
| Ki67 | Biolegend | 652402 |
| CD1 | Abcam | ab95734 |
| LY6C | Abcam | ab54223 |
| CX3CR1 | Fluidigm | 3164023B |
| FOXP3 | Fluidigm | 3165024A |
| IL4 | Fluidigm | 3166003B |
| CD169 | R＆D | MAB5610 |
| CCR7 | R＆D | MAB3477 |
| GFAP | Abcam | ab218309 |
| CD80 | Fluidigm | 3171008B |
| CD86 | R＆D | MAB741 |
| _Granzyme_B | Fluidigm | 3173006B |
| LY6G | Fluidigm | 3174008B |
| MHCII | R＆D | FAB6118A |
| B220 | Fluidigm | 3176002B |
| CD11C | Fluidigm | 3209005B |
| CD45-FITC | Biolegend, | 103108 |
| CD3- PerCP/cy5.5 | Biolegend | 100218 |
| CD4-APC/cy7 | Biolegend | 100526 |
| CD11b-PE/cy7 | Biolegend | 101216 |
| CD69-APC | Biolegend | 104514 |
| Alexa Fluor488/donkey anti-rabbit IgG | Invitrogen | A21207 |
| Alexa Fluor 594/ donkey anti-rabbit lgG | Invitrogen | A32754 |
| Alexa Fluor 555/ donkey anti-Rat lgG | Invitrogen | A48270 |
| CD4 | CST | 25229S |
| NeuN | Abcam | ab177487 |
| mS1P1 | R&D | FAB7089B |
| TUNEL assay kit | Roche | 11684817910 |
| Collagenase IV | Solarbio | C8160 |
| Percoll | GE Healthcare | 17-0891-09 |
| Evans Blue | Sigma | E2129 |
| Mice Cytokine Array Panel A Kit | R&D | ARY006 |
| Trizol | Invitrogen | 15596026 |
| All-in-One First-Strand cDNA Synthesis SuperMix for qPCR(One-Step gDNA Removal) | TransScript | AT341 |
| TNF-α | CST | 11948 |
| IL-1β | Abcam | ab283818 |
| IFN-γ | R&D | 98139 |
| Β-actin | Abcam | Ab8244 |

**Figure S1 ISF from injured brain promotes brain T lymphocyte infiltration**

**A.** Schematic diagram of the stimulation of CLNs by brain injury products in non-TBI mice. **B-E.** The proportion and count of brain infiltrating CD4^+^T cells and CD4^+^CD11b^+^T cells (n=8). **F.**The proportion of brain CD4^+^CD11b^+^T cells among CD3^+^T cells (n=8). **G.**The CD11b MFI on brain CD4^+^T cells between sham+ISF and Ligation+ISF mice at 7dpi (n=6). All data are shown as mean ± SD. **p*< 0.05, **p< 0.01, ***p< 0.001, ****p< 0.0001. ISF, brain interstitial fluid; MFI, mean fluorescence intensity; CLNs, deep cervical lymph nodes; dpi, day post-injury; TBI, traumatic brain injury.

**Figure S2 ISF from injured brain promotes CLNs CD4^+^T-lymphocyte release**

1. **B.** The proportion and count of CD4^+^CD11b^+^T cells in the CLNs and blood of sham mice (n=6). **C.** The count of clusters 20, 22, and 24 per 10^6^ cells at different time points. **D.** The proportion of CD3^+^T cells, CD4^+^T cells, and CD4^+^CD11b^+^T cells on the CD45^+^ cells in the CLNs of TBI and Ligation+TBI mice at 7 dpi (n=8). **E.** Display of Evans Blue in the ligated CLNs on the 7th day after ligation. **F.** The representative image of ligated CLNs after adding fluorescein at 3rd or 7th day after ligation. Scale 50 µm. **G.** Mean fluorescence intensity in the CLNs on the 3rd and 7th day after ligation (n=5). **H.** The proportion of CD4^+^CD11b^+^T cells on the CD45^+^ cells in the TBI mice blood at 1, 3, 7 dpi and cervical lymphadenectomy +TBI mice. **I.** The count of CD4^+^CD11b^+^T cells in the blood at 7 dpi between TBI and cervical lymphadenectomy +TBI mice. **J-K** The proportion of CD4^+^T and CD4^+^CD11b^+^T among CD45^+^ cells between sham+ISF and Ligation+ISF mice in the CLNs (n=5-6). **L-M.** The relative CLNs S1PR1 MFI on CD4^+^T, and CD4^+^CD11b^+^T cells divided by the S1PR1 MFI of sham mice (n=4-6). All data are shown as mean ± SD. *p< 0.05, **p< 0.01, ***p< 0.001, ****p< 0.0001. TBI, traumatic brain injury; MFI, mean fluorescence intensity; CLNs, cervical lymph nodes.

**Figure S3. CD4^+^CD11b^+^ T cells exacerbate brain edema.**

1. Edema lesion of the injured brain with 9.4T MRI. **B.** The statistical volumetric histogram of edema (n=6). All data are shown as mean ± SD. *p< 0.05, **p< 0.01, ***p< 0.001, *****p*< 0.0001. TBI, traumatic brain injury; CLNs, deep cervical lymph nodes; MRI, **Magnetic Resonance Imaging.**
